# Recent Advances in Brain Cancers

**DOI:** 10.3390/ijms26125755

**Published:** 2025-06-16

**Authors:** Pierre-Aurélien Beuriat

**Affiliations:** 1 Department of Pediatric Neurosurgery, Hôpital Femme Mère Enfant, Hospices Civils de Lyon, 69002 Lyon, France; pierre-aurelien.beuriat@chu-lyon.fr; 2 Rockfeller School of Medicine, Claude Bernard University, 69008 Lyon, France

Brain tumors in humans remain a challenge for clinicians and researchers. However, their overall prognosis has improved in recent decades owing to a better understanding of “why” these tumors occur and the “how” we can treat them. Nevertheless, they often continue to represent a fatal disease. It is crucial to enhance our knowledge of the cellular characteristics, development, and response of these tumors to treatments, as well as our knowledge of the normal phenomena surrounding tissues. This would enable us to improve patients’ survival and quality of life.

In this Special Issue, multiple aspects related to the biology and treatment of brain tumors are discussed.

The first study (contribution 1) investigated bone morphogenetic protein (BMP) 4, a potential therapeutic glioblastoma (GBM) agent with the ability to induce senescence in GBM cells. Primary GBM cultures were treated with BMP4 and analyzed for senescence markers, including cell enlargement, p21 expression, senescence-related gene enrichment, and senescence-associated-β-galactosidase activity. A p21 knockout model was used to determine its role in BMP4-induced senescence, and sensitivity to the senolytic agent navitoclax was evaluated. BMP4 induced senescence in the GBM cultures, particularly in mesenchymal (MES)-like GBM cells with high baseline p21 levels. The knockout of p21 nearly abolished BMP4-induced senescence, maintaining cell size and proliferation. Furthermore, navitoclax effectively eliminated BMP4-induced senescent cells through apoptosis, while sparing cells with normal p21 expression. These findings highlight BMP4 as an inducer of p21-dependent senescence in GBM, particularly in MES-like cells, and clarifies BMP4’s dual roles in differentiation and senescence, emphasizing their context dependence. Considering the strong association between MES-like cells and therapy resistance, their heightened susceptibility to senescence may aid in developing targeted therapies for GBM and potentially other cancers with similar cellular dynamics.

A second study (contribution 2) described the expression of CSF3R mRNA in human gliomas and their association with patient prognosis, as assessed by overall survival (OS). The authors found that the levels of CSF3R/CD114 transcripts are higher in a few different types of gliomas, namely astrocytoma, pilocytic astrocytoma, and GBM, in comparison to non-tumoral neural tissue. They also observed that a higher expression of CSF3R/CD114 in gliomas is associated with poorer outcomes, as measured by a shorter OS. Their findings provide early evidence suggesting that CSF3R/CD114 could be employed as a prognosis marker of OS in patients with GBM.

A third study (contribution 3) aimed to identify the metabolomic signatures associated with the gliomagenesis pathway (IDH-mutant or IDH-wt) and tumor grade of diffuse gliomas (DGs) according to the 2021 WHO classification of frozen samples, and to evaluate the diagnostic performances of these signatures in tumor samples that are formalin-fixed and paraffin-embedded (FFPE). An untargeted metabolomic study was performed using liquid chromatography/mass spectrometry on a cohort of 213 DG samples. Logistic regression with LASSO penalization was used on the frozen samples to build classification models in order to identify IDH-mutant vs. IDH-wildtype DG and high-grade vs. low-grade DG samples. The metabolite 2-Hydroxyglutarate (2HG) was utilized to predict the mutational status of IDH, and aminoadipic acid (AAA) and guanidinoacetic acid (GAA) were significantly associated with grade. The diagnostic performances of the models were 82.6% AUC, 70.6% sensitivity and 80.4% specificity regarding the ability of 2HG to predict the IDH status, and 84.7% AUC, 78.1% sensitivity and 73.4% specificity regarding the ability of AAA and GAA to predict grade from FFPE samples. Thus, this study revealed that AAA and GAA are two novel metabolites of interest in DG and that metabolomic data can be employed in the classification of DG, both in frozen and FFPE samples.

A fourth study (contribution 4) used a large Medulloblastoma (MB) tumor dataset to examine the gene expression of Neuropilin-1 (NRP1) in different molecular subgroups and subtypes of MB. The authors found that the overall expression of NRP1 was widespread across MB samples. Tumors in the sonic hedgehog (SHH) subgroup showed significantly higher NRP1 transcript levels in comparison with the tumors in Group 3 and Group 4, with SHH samples belonging to the α, β, Δ, and γ subtypes. When all MB subgroups were combined, lower NRP1 expression was associated with a significantly shorter overall survival (OS). Further analysis showed that a reduction in the expression of NRP1 was related to a poorer OS, specifically in the MB subgroups SHH and Group 3 MB. Their findings indicated that patients with SHH and Group 3 tumors with a lower expression of NRP1 in MB exhibit a worse prognosis; this highlights the need for the subgroup-specific investigation of the role of NRP1 in MB.

A fifth paper (contribution 5) reviewed the embryological origins of chordomas, molecular markers such as brachyury, and the genetic alterations promoting pathogenesis.

A sixth paper (contribution 6) assessed the use of liquid biopsy in the management of brain tumors. The authors reviewed the current research and the challenges associated with implementing liquid biopsy techniques for advancing meningioma patient care.

Finally, the last paper (contribution 7) investigated the dysregulation of the BMP pathway, which is one the major signaling pathways implicated in embryonic development, ontogeny and homeostasis, and its role in Diffuse Midline Gliomas (DMG), especially in Diffuse Intrinsic Pontine Gliomas (DIPG). The authors reviewed recent findings regarding the involvement of BMP pathway activation in these tumors, placing their appearance in a developmental context. They also suggested that targeting the oncogenic synergy that results from the activation of this pathway in an H3K27M context could offer novel therapeutic approaches based on targeting treatment-resistant cell states.

By bridging fundamental research and clinical applications, the contributions in this Special Issue offer valuable insights into current brain cancer research. Future studies should aim to enhance our understanding of brain cancer biology and its response to treatment, explore minimally invasive methods of diagnosis, and standardize clinical protocols in order to improve patients’ survival and quality of life.

